# Variables Connecting Parental PTSD to Offspring Successful Aging: Parent–Child Role Reversal, Secondary Traumatization, and Depressive Symptoms

**DOI:** 10.3389/fpsyt.2019.00718

**Published:** 2019-10-15

**Authors:** Yaakov Hoffman, Amit Shrira

**Affiliations:** The Interdisciplinary Department of Social Sciences, Bar-Ilan University, Ramat-Gan, Israel

**Keywords:** Holocaust, intergenerational transmission, parental posttraumatic stress disorder, parent–child role reversal, successful aging

## Abstract

The effects of parental trauma on offspring of Holocaust survivors (OHS) are debated in the literature. Recently, scholars suggested that it may be more productive to ask when and *via* which mechanisms such effects are observed. Following, the current study examines if parental Holocaust-related posttraumatic stress disorder (PTSD) symptoms are linked with the aging processes of their middle-aged offspring. Beyond this association, we also suggested a putative mediation path, indicating three underlying mechanisms by which parental trauma lingers on: perceived parent–child role reversal, secondary traumatization, and depressive symptoms. Using a convenience sample of 682 community-dwelling participants, comprising 341 older adult parent–middle-aged offspring dyads (*M* age = 81.71 and 54.58 for parents and offspring, respectively) to address this issue. Parents reported PTSD with the valid measure of PTSD Checklist for Diagnostic and Statistical Manual of Mental Disorders, fifth edition. OHS reported perceived parent–child role reversal, secondary traumatization, depressive symptoms, and completed indices of successful aging. Based on parents’ reports, we divided the parent–offspring dyads into three groups: OHS whose parents had probable PTSD (*n* dyads = 43), OHS whose parents did not have PTSD (*n* dyads = 161), and comparison with parents who did not undergo the Holocaust (*n* dyads = 137). Findings reveal that OHS with parents suffering from probable PTSD aged less successfully than comparisons. Serial mediation analyses validated the aforementioned putative pathway (perceived parent–child role reversal, secondary traumatization, and depression) linking parental PTSD with offspring successful aging. Our findings are discussed through a vignette depicting a fictional OHS character. These underlying mechanisms suggest that different types of interventions, each geared towards a specific mechanism, may mitigate the lingering effect of parental PTSD on diminished OHS successful aging.

## Introduction

A fair amount of earlier evidence suggests that mass trauma exposure, such as genocide, may shape late-life physical [e.g., (1–3)] and psychological (4, 5) morbidity in survivors. Admittedly, less is known about how parental exposure to genocide may affect the aging process of subsequent generations which were not directly exposed [see (6) for exception]. Accordingly, the goal of the current study is twofold: first, to examine whether offspring of Holocaust survivors’ (OHS) aging is associated with parental trauma and posttraumatic stress disorder (PTSD), namely, tying parental trauma to offspring’s successful aging; second, to assess potential mediators that may link parental trauma and PTSD to OHS’s successful aging. These mediators include the parent–child role reversal common in Holocaust survivor families, whereby the child assumes a parental role as explained below, as well as secondary traumatization and depressive symptoms experienced by the OHS. These variables are hypothesized to serially mediate the association between parental PTSD and the offspring’s successful aging.

### Trauma and Successful Aging

The notion that parents’ trauma transmits into offspring has been debated (e.g., 7–9). Using a meta-analysis to examine 32 studies on this topic did not reveal an overall effect of OHS being more impaired on psychosocial functioning (10). Still, this study did find indications of greater distress among OHS relative to comparisons if they experienced stressful events. For example, OHS who coped with breast cancer reported greater distress relative to comparisons (those whose parents did not undergo the Holocaust) patients (11). Recently, a literature review (12) revealed that half the studies addressing transmission of trauma to OHS found some indications of more psychopathology in OHS participants; yet, these authors noted that those studies were methodologically inferior to the studies which did not find such effects. In both middle-aged and older OHS, impaired physical health was noted [(13, 14); but see (15), who did not find such results].

In light of the mixed evidence regarding mental and physical health of OHS, researchers claim that efforts should shift from focusing on “if OHS are more vulnerable?,” to questions addressing the mechanisms and conditions, under which transmission may be observed. For example, in which families should we expect to see trauma transmission, and by which mechanisms should transmission transpire (8, 16)? Following, it may be that intergenerational transmission of trauma is not automatic, nor is it an unavoidable outcome of parental trauma; rather, it may stem from an unresolved attempt by parents to cope with their own trauma.

Aligned with this idea, parental PTSD has been generally associated with an increased risk for psychopathology in young offspring (17, 18). OHS also showed increased vulnerability to PTSD and other psychiatric disorders, especially when they perceived both their parents to have PTSD (19) or perceived one of the parents to have a negative parental style (16), meaning parents characterized as being stuck in the trauma or those who are numb and emotionally detached. It remains to be resolved whether and how parental PTSD is linked with the aging process of OHS.

### Secondary Traumatization Mediates Parental PTSD–Offspring’s Successful Aging Link

The term secondary traumatization is defined by manifestation of PTSD symptoms in cases where there was no direct exposure, e.g., if an OHS would have nightmares about parental Holocaust experiences (20, 21).

In Shrira et al. (6), the link between parental PTSD–offspring’s successful aging was mediated by OHS’s secondary traumatization. This finding is most important for two reasons: first, not much is known with regard to secondary traumatization in OHS [for exceptions, see (22, 23)]. Second, although many studies have linked PTSD to impaired health and premature death (24–26) and negative aging evaluations (27), secondary traumatization is not often considered in the aging context. In the next two sections, we respectively address two additional potential mediators of the association between parental PTSD (emerging from long-term trauma) and offspring’s successful aging.

### Parent–Child Role Reversal

Initial data reveal elevated secondary traumatization in a minority of OHS who reported faulty parental communication of trauma, i.e., parental trauma was communicated in an intrusive manner [cf. (28)]. Their secondary traumatization level in turn was related to more impaired health problems and negative perceptions regarding aging (29). There are several documented detrimental parenting styles that some Holocaust survivors (HS) have been known to engage in, e.g. HS parents becoming overprotective, viewing the world as a place fraught with danger, such as allowing their children to go on an organized field trip (30, 31). In other cases, the parent who has not adequately dealt with their own trauma may reject their children. Yehuda et al. (32) found that the more an OHS felt rejected as a child by their parents, the greater the trauma transmission was. The third and perhaps most detrimental parenting style is that of role reversal, namely, the parental child, where the child assumes the role of the parent—who is too emotionally frail to function as a parent and who also has too many unmet needs (33). The parents’ psychopathological need for an attachment figure, due to their own arrested attachment resulting from childhood exposure to genocide, renders their own child, their “parent” (30, 31). Such OHS may feel burdened by being charged with taking care of parents’ emotions and well-being at the expense of their own attachment and development. Some HS, particularly those who are more affected by their past trauma, may indirectly convey their emotional susceptibility to OHS, causing them to be the “parent,” who is now worrying about their well-being [see (23, 34)]. We thus hypothesize that parental PTSD may lead to role reversal, which will in turn lead to higher secondary traumatization [e.g., (23)]. Below, we address how this path of parental PTSD *via* role reversal, *via* secondary traumatization, will lead to depression.

### Secondary Traumatization and Depression

Although it is known that PTSD and depression overlap often, and that such a comorbid condition is often qualitatively different (35), depression is often ignored. Depression is also frequently ignored with regard to the issue of secondary traumatization, although we know that there are strong associations between the two conditions (36). This lacuna also exists with regard to the Holocaust literature (37, 38). Nevertheless, it is very likely that parental PTSD, leading to OHS role reversal, which would putatively perpetuate a higher secondary traumatization level, would cumulate in high levels of depressive symptoms. In turn, suffering from depressive symptoms would, by definition, impair one’s level of successful aging, as noted below.

### Successful Aging

Successful aging may be addressed in several manners. The seminal successful aging operationalization by Rowe and Kahn (39) is based on the tenet that disease, disability, and decline may not be inevitable aging processes. Successful aging is thus described as the combination between a lack of disease and disability along with an active engagement with life. Some critique this position, as it may include very few older people who are able to maintain high levels of functioning (40). A contemporary view suggests moving from a binary definition of successful aging (i.e., successful vs. unsuccessful aging) to a continuous measure, as even when aging may be associated with limitations in one aspect of functioning, persons may perform relatively well in other domains (41). Following, we operationalize successful aging as a continuous, multidimensional construct that incorporates several indices [cf. (42)].

### Summary and Hypotheses

To recapitulate, we assessed the relationship of parental PTSD with the primary outcome of successful aging, as well as with the secondary outcomes of parent–child role reversal, secondary traumatization, and depressive symptoms. We propose a model in which parental PTSD relates to role-reversal (parental child) among OHS. Role reversal should associate with higher levels of secondary traumatization in OHS, which in turn should be positively associated with their depressive symptoms. Finally, higher depressive symptoms should associate with less successful aging in OHS.

More specifically, we hypothesized that OHS with parental PTSD will present less successful aging relative to comparisons (i.e., those whose parents were not exposed to the Holocaust), whereas OHS without parental PTSD will age as successfully as comparisons. Second, we hypothesized that the association of parental PTSD with offspring successful aging will be serially mediated *via* parenting (role reversal), OHS’s secondary traumatization level, and level of depressive symptoms.

## Material and Methods

### Participants

A convenience sample included 682 community-dwelling participants, who who comprised 341 dyads, of parents and adult offspring. All parents were Jewish of European origin born before 1945. Offspring were born after 1945 and had two parents who were alive during World War II. HS and their offspring included 204 dyads, and comparison parents without a Holocaust background and their offspring included 137 dyads. Holocaust background was determined by parents’ presence under Nazi or pro-Nazi occupation or domination during World War II.

Dyads were next divided according to probable parental PTSD (for more details see the Measures section). There were 43 Holocaust dyads with a parent suffering from probable PTSD, 161 Holocaust dyads with a parent without PTSD, and 137 comparison dyads (all of them with parents without PTSD).

**Table 1** presents the background characteristics of the study groups. HS with or without PTSD were older than comparison parents. HS with probable PTSD had lower education level and rated their economic status as lower than both other groups. The groups did not significantly differ in parental gender and marital status. OHS whose parents had probable PTSD were older relative to comparison offspring. The offspring groups did not significantly differ in any of the other background characteristics.

**Table 1:** Background characteristics of the study groups.

	Holocaust survivors with probable PTSD dyads	Holocaust survivors without PTSD dyads	Comparison dyads	Comparison tests
n	43	161	137	
Parents				
Mean age (SD)	83.58^a^(5.12)	82.63^a^(5.84)	80.05^b^(6.10)	F(2,338) = 9.68, p < 0.0001, η^2^ = 0.05
Gender (%)				χ^2^(2) = 1.80, p = 0.40
Woman	72.1	62.1	67.2	
Man	27.9	37.9	32.8	
Education (%)				χ^2^(4) = 32.99, p < 0.0001, ϕ_c_ = 0.31
Below high school	67.4	44.4	25.2	
Full high school	16.3	24.4	21.5	
Above high school	16.3	31.3	53.3	
Marital status (%)				χ^2^(8) = 11.24, p = 0.18
Married	39.5	46.9	55.5	
Widowed	58.1	45.6	39.4	
Divorced	0.0	5.6	2.2	
Single	2.3	0.6	0.7	
Partner	0.0	1.3	2.2	
Mean self-rated economic status (SD)	3.07^a^(0.70)	3.51^b^(0.77)	3.63^b^(0.86)	F(2,335) = 8.03, p < 0.0001, η^2^ = 0.04
Offspring				
Mean age (SD)	56.20^a^ (5.68)	55.06^a,b^ (6.07)	53.50^b^(5.57)	F(2,338) = 4.56, p = 0.01, η^2^ = 0.02
Gender (%)				χ^2^(2) = 0.63, p = 0.72
Woman	67.4	62.1	65.7	
Man	32.6	37.9	34.3	
Education (%)				χ^2^(4) = 1.80, p = 0.77
Below high school	2.3	1.9	4.4	
Full high school	20.9	18.9	19.7	
Above high school	76.7	79.2	75.9	
Marital status (%)				χ^2^(8) = 8.36, p = 0.39
Married	83.7	84.7	85.3	
Widowed	4.7	0.6	2.9	
Divorced	9.3	11.5	5.9	
Single	0.0	1.9	2.9	
Partner	2.3	1.3	2.9	
Mean self-rated economic status (SD)	3.69(0.80)	3.84(0.80)	3.97(0.84)	F(2,337) = 2.27, p = 0.10

When the F test was significant, we performed a post hoc Bonferroni test to assess main effect differences. The superscript letters (a and b) represent means that significantly differ from each other in the post hoc tests (i.e., if one mean is marked with “a” and another mean is marked with “b,” these two means are significantly different from each other).

In the total sample, 12.6% of parent–offspring dyads were father–son dyads, 22.0% were father–daughter dyads, 23.2% were mother–son dyads, and 42.2% were mother–daughter dyads. The ratio of the dyad types did not significantly differ across the three study groups, *χ* ^2^(6) = 3.20, *p* = 0.78.

Among HS, the median year of emigrating to Israel was 1949, and median age at the time of immigration to Israel was 19. Holocaust-related experiences (e.g., being in a concentration camp, work camp, ghetto, hiding, living with partisans, having been exposed to hunger, extreme weather conditions, and extreme physical abuse) were documented among HS. Compared to survivors without PTSD, a significantly greater number of survivors with probable PTSD were in work camps [41.9% vs. 19.3%, *χ* ^2^(1) = 9.50, *p* = 0.002], exposed to hunger [37.2% vs. 20.5%, *χ* ^2^(1) = 5.19, *p* = 0.02), extreme weather conditions [34.9% vs. 19.3%, *χ* ^2^(1) = 4.74, *p* = 0.02], and physical abuse during the Holocaust [16.3% vs. 5.0%, *χ* ^2^(1) = 6.37, *p* = 0.01]. Significantly more survivors without PTSD reported to have been in hiding than survivors with probable PTSD [47.8% vs. 23.3%, *χ* ^2^(1) = 8.37, *p* = 0.004].

### Measures

*Background characteristics*. Background characteristics were completed by all respondents and included age, gender, education, and marital status. Education was rated on a scale from “no formal education” (1) to “academic degree” (6). Self-rated economic status was rated with a single item on a scale from “not good at all” (1) to “very good” (5).

*PTSD symptoms.* PTSD symptoms were rated by all parents *via* the PTSD Checklist for Diagnostic and Statistical Manual of Mental Disorders, fifth edition (43). This questionnaire is a 20-item measure of PTSD symptoms as appearing in the Diagnostic and Statistical Manual of Mental Disorders, fifth edition (44). HS were instructed to refer to their experiences during the Holocaust, whereas comparison parents were instructed to refer to the most traumatic event that had happened to them. Most comparison parents referred to a sudden death of a close person (32.3%) or to a life-threatening illness or disability that happened to a close person (19.4%). Others referred to being diagnosed with a life-threatening illness (8.1%), undergoing (or being injured in) war, combat or terrorist attack (9.6%), surviving a severe accident (6.5%), being physically attacked (4.8%), or undergoing other life-threatening events (e.g., natural disaster, experiencing abuse, etc., 19.3%).

For each symptom, parents were asked to choose their response on a 5-point Likert scale from 1 (*not at all bothered*) to 5 (*extremely bothered*) when referring to the last month. The PTSD symptom score was the sum of ratings. Higher scores indicated higher levels of PTSD symptoms. The PTSD Checklist for Diagnostic and Statistical Manual of Mental Disorders, fifth edition was found to have excellent convergent and discriminant validity (45). The Hebrew version of the scale was previously used (6). Cronbach’s *α* coefficient for this sample was 0.91. Probable PTSD was determined by a cut-off score of 33 or higher (cf. 45).

*Perceived parental rearing behaviors*. Offspring completed the Perceived Parental Rearing Behavior Questionnaire (46). The questionnaire includes 20 items examining perceived parenthood in four areas. The advantage of a self-report measure is that, frequently, the subjective experiences are more influential than the so-called objective reality (46). Offspring were instructed to refer to the parent who participated in the study. For each behavior, offspring were asked to choose their response on a 5-point Likert scale from 1 (*not at all*) to 5 (*all the time*) when referring to how the parent behaved towards them during their childhood. The behavior score was the sum of ratings in each scale. The scales included role reversal (seven items; e.g., “I felt like a parent to my parent”), affection (seven items; e.g., “My parent showed me that s/he loved me”), punishing (three items; e.g., “My parent hit me”), and overinvolvement/protection (three items; e.g., “My parent was too involved in my life”). Higher scores indicated higher frequency for each of the behaviors. Cronbach’s *α* coefficient was 0.71, 0.88, 0.66, and 0.70, for role reversal, affection, punishing, and overinvolvement/protection scale, respectively. The original questionnaire was developed in Hebrew.

*Secondary traumatization*. Offspring completed the modified Secondary Trauma Questionnaire (21). They rated the frequency in which they experienced 18 symptoms of distress, due to traumatic events experienced by their parent, using a scale ranging from 1 (*never or rarely*) to 5 (*very often*). Participants were asked to specifically refer to the parent who participated in the study. OHS were asked to relate to the Holocaust as the traumatic event, and comparisons were instructed to refer to the traumatic event reported by their parents (the students informed them of that event before they began to complete the questionnaire). The final score was based on the sum of answers. Higher scores reflect higher secondary traumatization. Cronbach’s *α* was 0.91. Previous studies have used the Hebrew version of this measure [e.g., (29, 47)].

*Depressive symptoms*. Offspring completed the depression subscale derived from the 18-item Brief Symptom Inventory (BSI-18) (48). We computed the mean of six items rated on a scale ranging from 0 (*not at all*) to 4 (*very much*) with a Cronbach’s *α* of 0.86. Previous studies have used the Hebrew version of this measure [e.g., (6)].

*Successful aging*. Offspring completed several indices of successful aging including chronic medical conditions, disability, somatic symptoms, and a global assessment of health (self-rated health).

Chronic medical conditions were assessed by a sum of 11 listed illnesses that participants reported to have been diagnosed with by a physician. The illnesses consisted of heart disease, high blood pressure, high cholesterol, stroke or cerebral vascular disease, diabetes or high blood sugar, chronic lung disease, such as chronic bronchitis or emphysema, asthma, arthritis (including osteoarthritis or rheumatism), osteoporosis, cancer or malignant tumor, and Parkinson’s disease.

Disability was measured by asking respondents to rate difficulties in preforming five functional activities [adapted from (49)] including stooping, kneeling, or crouching, reaching or extending arms above shoulder level, pulling or pushing heavy objects, lifting or carrying heavy weights, and picking up a small coin from a table. Each activity was rated on a scale from 1 (*not difficult to perform at all*) to 4 (*extremely difficult to preform*). The final score was based on the average of answers. Higher scores reflect higher disability. Cronbach’s *α* was 0.70. Previous studies have used the Hebrew version of this measure [e.g., (29)].

Somatic symptoms were assessed using the somatization subscale derived from the 18-item Brief Symptom Inventory (BSI-18) (48). We computed the mean of six items rated on a scale ranging from 0 (*not at all*) to 4 (*very much*) with a Cronbach’s *α* of 0.76. Previous studies have used the Hebrew version of this measure [e.g., (6)].

Self-rated health was rated on a scale from 1 (*very good*) to 5 (*not good at all*) (50).

In order to compute the overall successful aging score, the four scores were standardized (medical conditions, disability, somatic symptoms, and the reverse-coded self-rated health score), and then, the standardized scores were averaged. Subsequently, the averaged standardized score was multiplied by −1, so that high scores will reflect greater successful aging [cf. (6, 51)].

### Procedure

Undergraduate student from a yearly seminar were instructed to recruit eligible participants available in their surroundings. The seminar students were instructed how to approach the interviewees and respond to potential difficulties. This study was conducted across three yearly seminars, from January 2014 until April 2018. The seminar students requested participants to take part in a study, which aimed to examine how families cope with difficult life events. Participants read and signed an informed consent form, which also noted that the questionnaire included queries regarding aging, death, various difficult life events, and the Holocaust. Following that, participants (mostly offspring) accessed an online questionnaire *via* a link sent to them. The students interviewed participants (mostly parents) who could not complete the online questionnaire themselves. Participants were interviewed, predominantly in the urban areas around Haifa (12.8% of HS living in Israel), Tel Aviv (12.0%), and Jerusalem which (11.3%), areas with the highest HS prevalence in Israel^1^ in their homes or other places convenient to them. The study received approval by an ethic review committee in Bar-Ilan University on November, 2014.

### Data Analysis

Group differences in perceived parental rearing behaviors, secondary traumatization, depressive symptoms, and successful aging were assessed with a series of univariate analyses of covariance (ANCOVAs). As parental age and education level, as well as offspring age, significantly differed between the groups, these variables were controlled for. We also controlled for parental and offspring gender, as gender may potentially moderate intergenerational transmission (19).

The PROCESS macro (52) was applied to test the hypotheses regarding the serial mediation effects. The multicategorical independent variable (study groups) was coded into two dummy indicator variables, *D*_1_ and *D*_2_, denoting Holocaust exposure without PTSD and with probable PTSD, respectively, and leaving the comparisons as the reference group. To predict offspring successful aging, we used perceived parental rearing behavior (the ones found to significantly^1^ differ between the groups), secondary traumatization, and depressive symptoms as three mediators, *M*_1_, *M*_2_, and *M*_3_, respectively. Possible mediation paths were assessed in a serial mediation analysis using a bias-corrected bootstrap with 5,000 resamples. The serial mediation analysis controlled for all the abovementioned covariates.

## Results

### Group Differences in Main Study Variables

**Table 2** presents the results of the ANCOVAs comparing the groups on successful aging, perceived parental rearing behaviors, secondary traumatization, and depressive symptoms.

**Table 2:** Results of univariate analyses of covariance comparing groups on successful aging, perceived parental rearing behaviors, secondary traumatization, and depressive symptoms.

Variable	OHS with parental PTSD	OHS without parental PTSD	Comparisons without parental PTSD			
	*M* (*SD*)	*M* (*SD*)	*M* (*SD*)	*F*	*p*	*η* ^2^
Successful aging	−0.26 (0.92)^a^	−0.09 (0.70)^a^	0.20 (0.56)^b^	8.80	<0.0001	0.051
Role reversal	2.59 (0.84)^a^	2.20 (0.73)^b^	2.06 (0.74)^b^	6.83	0.001	0.041
Affection	3.21 (1.02)	3.10 (1.08)	3.12 (1.17)	0.16	0.849	0.001
Punishing	2.10 (0.74)	1.91 (0.69)	1.90 (0.74)	1.21	0.299	0.007
Over-involvement	3.54 (0.77)	3.33 (1.00)	3.27 (0.95)	1.17	0.309	0.007
Secondary traumatization	32.37 (11.22)^a^	26.96 (9.90)^b^	24.54 (8.62)^b^	9.32	<0.0001	0.055
Depressive symptoms	1.61 (0.77)^a^	1.35 (0.57)^b^	1.22 (0.46)^b^	6.98	0.001	0.042

n is 43, 161, and 137 for OHS with parental PTSD, OHS without parental PTSD, and comparisons, respectively. Means are adjusted for parental and offspring age, parental and offspring gender, parental education level, and self-rated economic status. Means that do not share letters significantly differ from each other in a post-hoc Bonferroni test.

The groups significantly differed in successful aging, role reversal, secondary traumatization, and depressive symptoms. Bonferroni *post hoc* tests showed that OHS with or without parental PTSD had lower successful aging score than comparisons. OHS with parental PTSD also reported higher role reversal, secondary traumatization, and depressive symptoms compared with both other groups. The groups did not differ in perceived parental affection, punishing, or overinvolvement.

Supplementary analyses assessed the effect of paternal vs. maternal PTSD. OHS with parental PTSD were thus divided into two subgroups: those with paternal (*n* = 12) and maternal PTSD (*n* = 31). In ANCOVAs comparing the four groups, i.e, OHS with paternal PTSD, maternal PTSD, OHS without parental PTSD, and comparisons, we controlled for all abovementioned covariates except for parental gender. The groups differed in successful aging, *F*(3,326) = 6.00, *p* = 0.001, *η* ^2^ = 0.052. Bonferroni post-hoc tests showed that all three OHS groups (with paternal or maternal PTSD and without parental PTSD) reported lower successful aging score than comparisons. The groups further differed in role reversal, *F*(3,322) = 4.81, *p* = 0.003, *η* ^2^ = 0.043, and secondary traumatization, *F*(3,318) = 6.40, *p* < 0.0001, *η* ^2^ = 0.057. Both OHS with paternal or maternal PTSD reported higher role reversal and secondary traumatization than comparisons. The groups did not differ in perceived parental affection, punishing, or overinvolvement (*F* < 1.10). Finally, the groups differed in depressive symptoms, *F*(3,321) = 4.64, *p* = 0.003, *η* ^2^ = 0.042. OHS with maternal PTSD reported higher depressive symptoms than comparisons.

### Serial Mediation Analysis Predicting Offspring Successful Aging

**Table 3** presents the findings from the serial mediation analyses (adjusted for covariates). Both parental exposure to the Holocaust (*D*_1_) and parental PTSD (*D*_2_) predicted lower successful aging score among offspring (*Y*). As both dummy variables were significant, the group difference reflected lower successful aging among OHS (both with and without probable PTSD) relative to comparisons. Parental PTSD explained 8% of the variance in successful aging. Moreover, parental PTSD (*D*_2_) predicted higher role reversal (*M*_1_). Parental PTSD explained 8% of the variance in role reversal. When both study group variables (*D*_1_ and *D*_2_) and role reversal (*M*_1_) were included as predictors of offspring secondary traumatization (*M*_2_), both parental PTSD (*D*_2_) and role reversal predicted higher secondary traumatization among offspring (*M*_2_) (explaining 24% of the variance in secondary traumatization). In addition, when both study group variables (*D*_1_ and *D*_2_), role reversal (*M*_1_), and secondary traumatization (*M*_2_) were included as predictors of offspring depressive symptoms (*M*_3_), both role reversal and secondary traumatization predicted higher depressive symptoms among offspring (*M*_3_) (explaining 34% of the variance in depressive symptoms). Finally, when study group variables (*D*_1_ and *D*_2_) alongside the three mediators—role reversal (*M*_1_), offspring secondary traumatization (*M*_2_), and offspring depressive symptoms (*M*_3_)—were included as predictors of offspring successful aging (*Y*), parental exposure to the Holocaust (*D*_1_), and offspring depressive symptoms (*M*_3_) significantly predicted less successful aging (*Y*) (explaining 34% of the variance in successful aging).

**Table 3:** Estimated unstandardized coefficients (coeff.) for the effect of study group (*D* *_1_* *and D*_2_) on offspring successful aging (*Y*), mediated by role reversal (*M*_1_), secondary traumatization (*M*_2_), and depressive symptoms (*M*_3_).

	Outcome									
	Perceived role reversal (*M*_1_)		Offspring secondary traumatization (*M*_2_)		Offspring depressive symptoms (*M*_3_)		Offspring successful aging (Y)			
							Mediated		Unmediated	
	Coeff.	*p*	Coeff.	*p*	Coeff.	*p*	Coeff.	*p*	Coeff.	*p*
Predictors										
Holocaust survivors without PTSD (*D*_1_)	0.11	0.22	1.93	0.08	0.05	0.39	−0.19	0.009	−0.28	0.0009
Holocaust survivors with probable PTSD (*D*_2_)	0.49	0.0006	5.27	0.002	0.11	0.20	-0.17	0.13	−0.45	0.0005
Perceived role reversal (*M*_1_)	—	—	5.36	<.0001	0.12	0.003	−0.05	0.35	—	—
Offspring secondary traumatization (*M*_2_)	—	—	—	—	0.03	<0.0001	−0.005	0.23	—	—
Offspring depressive symptoms (*M*_3_)	—	—	—	—	—	—	−0.56	<0.0001	—	—
*R* ^2^	0.08		0.24		0.34		0.34		0.08	
*F(df)*	3.23 (8,312)		11.11 (9,311)		16.37 (10,310)		14.37 (11,309)		3.64 (8,312)	
*P*	0.001		<0.0001		<0.0001		<0.0001		0.0005	

Analyses controlled for parent’s and offspring age, parental education level, and self-rated economic status, as well as parent’s and offspring gender.

The bootstrap analyses estimating the indirect effects of study group on offspring successful aging found three significant indirect effects. One indirect effect connected parental PTSD to less successful aging among offspring through a) higher perceived role reversal, which was then related to b) higher depressive symptoms [indirect effect = −0.03, 95% lower limit confidence interval (LLCI) = −0.08, 95% upper limit confidence interval (ULCI) = −0.005]. A second indirect effect connected parental PTSD to less successful aging among offspring through a) higher secondary traumatization, which was then related to b) higher depressive symptoms (indirect effect = −0.08, 95%LLCI = −0.17, 95%ULCI = −0.02). Finally, a third indirect effect connected parental PTSD to increased role reversal that was related to higher secondary traumatization among offspring, which was then related to higher depressive symptoms among offspring, which was finally related to less successful aging among offspring (indirect effect = −0.04, 95%LLCI = −0.08, 95%ULCI = −0.01).

**Figure 1:**
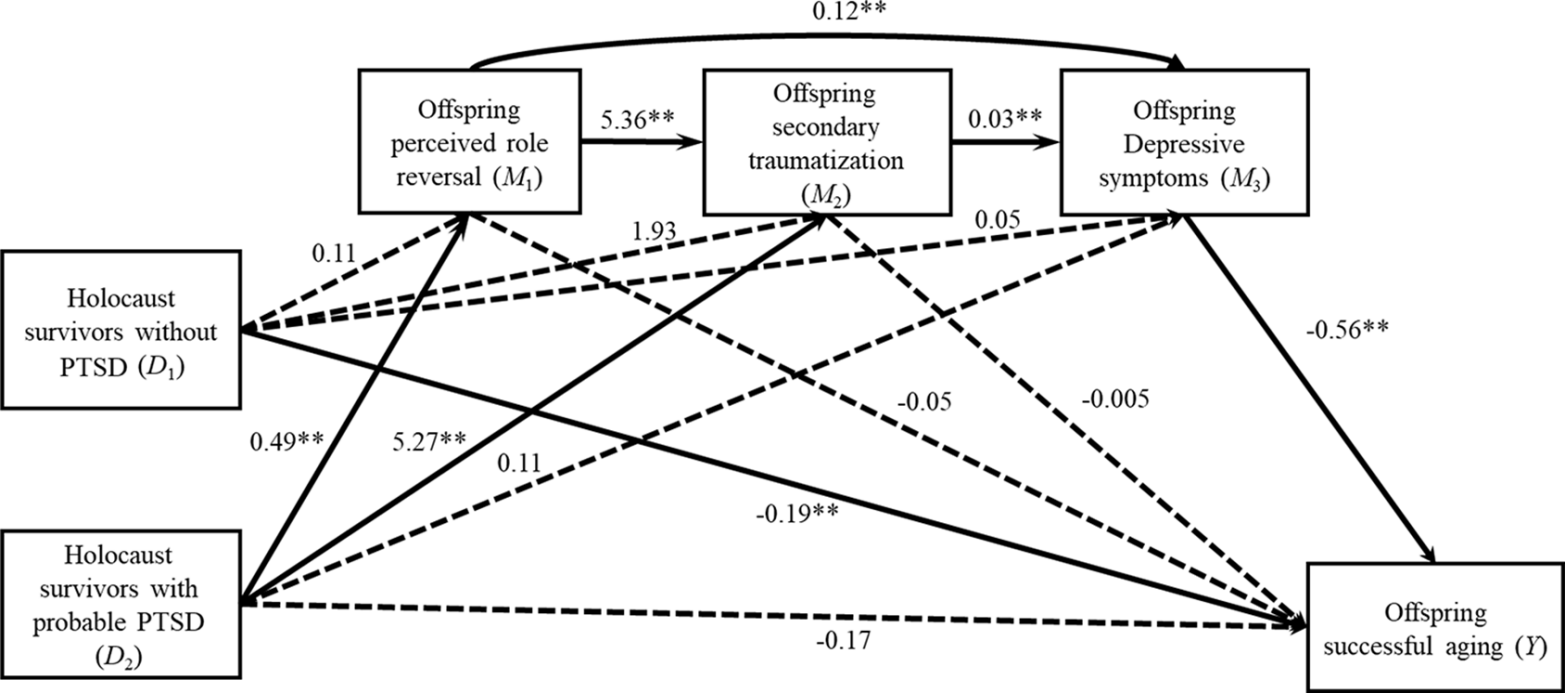
Offspring perception of role reversal, secondary traumatization, and depressive symptoms mediate the relationship between parental PTSD and offspring successful aging (controlled for parental and offspring age, parental education level, and self-rated economic status, and parent’s and offspring gender). Values refer to estimated unstandardized coefficients from the first four regression equations presented in **Table 3**. **p < 0.01.

**Figure 1** presents the indirect effects connecting parental PTSD to offspring successful aging.

## Discussion

This study has shown two main results. First, it revealed that the aftermath of parental PTSD lingers and is associated with successful aging of the middle-aged OHS, thereby suggesting intergenerational trauma transmission. Second, the current results support a pathway, which potentially reflects a putative underlying complex mechanism *via* which the aftermath of parental trauma lingers on more than seven decades later to affect OHS. Namely, parental PTSD can lead to a parent–child role reversal, which in turn may exacerbate secondary traumatization leading to OHS increased depressive symptoms, which in turn impairs successful aging. Each of these findings is discussed below.

The first finding suggests intergeneration transmission, echoing earlier scholars who called on reshifting the focus from asking—if intergenerational transmission of Holocaust trauma exists—to the question of, under what conditions does intergenerational transmission exist (8, 16)? The current study shows that it exists, at least when parents had PTSD (17, 18). This finding can be appended to those findings, which found effects of intergenerational transmission, at least under some conditions. As it seems that effects on offspring aging were not related to parental trauma exposure per se, but rather only to parental PTSD—these results as shown below (indirect pathway) seem able to resolve some of the aforementioned questions about the underlying mechanisms involved (8).

To the best of our knowledge, only one single paper has previously substantiated the OHS successful aging–parental PTSD link (6). Earlier works did not typically examine physical health among OHS (for exceptions, see 13–14, 15, 53). Even in those exceptions, parental PTSD was not addressed. Although lower health measures were obtained in OHS (with HS mothers) (13), such data emerged from a sample which may have been biased. Shrira et al. (14) revealed in a random sample that examined OHS vis-à-vis comparisons that OHS (especially OHS with two survivor parents) reported more medical issues, higher medication use, and increased physical symptoms. In other studies that applied nonselect samples, OHS were the same as comparisons in their physical morbidity (15, 53); note, however, that in both these studies, the OHS were relatively young.

### The Story of the Indirect Pathway

The main and critical finding, which was beyond the more direct track, suggests the underlying mechanisms linking parental PTSD and offspring aging. The mediation pathway tells a complex story that addresses the underling mechanisms involved in intergenerational transmission of trauma to OHS. These mechanisms can be seen as putative claims that may resolve Kellermann’s (8) question concerning how such trauma transmits.

The current results validated the entire pathway beginning from parental PTSD proceeding to parent–child role reversal, continuing to offspring secondary traumatization that potentially catalyze offspring depressive symptoms that finally may affect offspring aging. Although each individual link was established as reviewed in the introduction, herein, we address the integrative story. We do this with the aid of a fictional character called Sophia, who personifies a representative case of OHS whose parents suffered from PTSD. One of the first things Sophia said when asked to describe herself, was “that I would do anything to alleviate the suffering of my Mom. If anyone, and I mean anyone would hurt her, they will face my wrath.”

Sophia’s mother was in Auschwitz and had a tattooed number. She had PTSD and was frequently hypervigilant; thinking that Sophia was in danger, she also avoided Holocaust reminders such as going to a Holocaust museum or taking showers in a public place (e.g., beach), her arousal level when reminded about such issues rose dramatically, and as shown below, she also displayed negative alterations in cognitions. When Sophia was a baby, her mother was not always present emotionally; she felt her mother was “there”—thinking about her parents’ sibling who perished. She remembers her younger brother (Muli, named after her Father’s brother Shmuel) alternately being hugged very tight by her mother at times (as if to say I won’t let anything happen to you, literally afraid for his life, or perhaps her mother needed assurance that he was real) while at other times receiving less tactile affection. It was as if her mother expressed warmth but was hesitant/ambivalence towards hugging, perhaps being afraid to get too close, because it will bring only pain; it is a vulnerability to love. Sophia thinks that it was the same for her.

Incidentally, Sophia’s name was twist of a Yiddish name, named after her mother’s sister who perished. Sophia remembers from a very early age her mother saying that she Sophia is her mother’s victory over the Nazis. Sophia also remembers from a very young age her mother listening to a radio program for tracing lost relatives from the Holocaust, which was broadcasted every afternoon, where people called in giving names and places to ask if anyone knew what happened to them. She remembers the tension at home during this program being so “thick” along with the eerie “quiet.” Often, her mother after this program would be reclusive and withdrawn.

Her mother always worried and never believed anything a bureaucrat would say. Thus, for example, if the official at social security asked her to bring one document, she would literally panic and worry, looking for all documents that might be related. Sophia would calm her down and explain that she needed only the requested document. She would also often go with her mother on errands, where her presence as a reassuring figure, to calm her frantic mother down, was often needed.

One of Sophia’s earliest memories was to go into her father’s room when he was having a nightmare, screaming and shouting in Yiddish, a language she did not fully speak. In the beginning, she would sit there shaking. After a while, she felt it was her job to sit there, calm him down by either making soothing noises, stroking his arm, or bringing her father a cup of water. Incidentally, she felt that if she would rub off his tattooed number, she could make his pain disappear. When neighbors complained about the noise her father made at nights, they would come to her. Her father, a biochemist by training, worked in construction, rarely spoke, and in some ways much less dominant than her mother. Yet, there was much strength in his stoic but quiet demeanor.

Her mother, who worked in a bakery, tried her best to give her treats. Fridays were special, as Sophia would come after school to work in the bakery with her mother. At the age of 6, Sophia remembers a German tourist entering the bakery. Her mother literally lost it. She started screaming, that he should leave, people started stopping and asking what was wrong. A small-to-medium crowd of tens of people had already gathered. Her mother speaking German and screaming at this person, that he already did enough, she was thrusting her arm out to him with the tattooed number. At the end, the police arrived on the scene.

For Sophia, to obtain permission to go on a school field trip was no trivial manner. This is also an issue for her as a mom when her own child wants to go on a trip. Likewise, her staying out late at night as a teenager was often a point of profound strife. When a boy in her high school was killed in a terror attack, her mother who found her crying, said “crying will help bring him back?”

Indeed, it is very clear that Sophia’s parents loved her very much, and raised a family providing for them to their best ability. Yet, as they were suffering from PTSD, they may have been reliving the worst nightmares of the past and thus their diminished presence in the present. From Sophia’s case, one can obviously see that HS with PTSD, who may be living in the past, especially after being exposed to an infinite number of trauma reminders [cf. (54)], may be less available to care for the needs of their children leading to their greater susceptibility to developing psychological distress (55). A parent suffering from PTSD after experiencing massive trauma at a much younger age may not find adaptive ways to cope (30). Coping with such PTSD may demand many resources (56). When parental resources are lacking, as clearly in Sophia’s parents’ case, it may be conducive in creating a parent–child role reversal, as children sacrifice their own needs in order to try and satisfy the needs of their parents (57). Posttraumatic HS may also have themselves been robbed of forming secure attachment as they focused on survival during these formative years (30). Such a situation may lead to OHS becoming a “parent” to supply that protection and unconditional love towards their parents, who were themselves robbed.

One example driving this parent–child role reversal may be the recurrent thoughts experienced by parents. For example, a HS parent who experiences nightmares may be calmed down by the child who comes in every night, sits with the parent, and may bring them a cup of water. Other examples may relate to avoidance judgments the parents make (e.g., “Do not go on a field trip”), whereby the OHS may assume the role of psychotherapist, educator, parent—facilitating their “fitting in.”

Taking the above into consideration renders likely the probability of the “parental child” to experience more secondary traumatization. This is true not only because the parent exhibits PTSD (37), but rather also because the child has fewer resources and more difficulty with emotional regulation. Instead of being able to reframe, such persons may opt for avoidance of negative stimuli; while this may temporarily alleviate some pain, it will exacerbate the situation. This in turn may expedite the downhill spiraling of given situations, such as when being reminded of parental trauma, OHS might prefer a lower type of emotional regulation e.g.., avoidance. For example, when confronted with any reminder of parental PTSD (58), or any trauma reminder (54), e.g., seeing a child on television who wakes up from nightmares, such avoidance may in turn strengthen their levels of secondary traumatization.

Having elevated secondary traumatization would likely increase risk for developing depression (59). Often prolonged or chronic secondary traumatization is linked with depression (60). In addition, being depressed, especially as one ages, puts one in a frame of mind of a negative schema (Sophia as a mom being hesitant to let her own children go on a field trip), whereby it is likely that one will focus on losses as opposed to gains. This is important, as a balanced and adapted approach to losses and gains comprises an important concomitant of successful aging [e.g., (61)]. Accordingly, depression is associated with multiple adverse physical health outcomes (62). Therefore, similarly to the way depression mediates the effects of PTSD on health behaviors (e.g., physical inactivity and medication nonadherence) and health [cf. (62)], it may well mediate the effect of secondary traumatization on health.

Hitherto through the conglomerated persona of Sophia, we have tied the results with detailed description of what can transpire in some HS families. Still, it should be noted that Sophia’s case should not be considered as typical of all OHS. Indeed, most OHS function well to the same degree as comparisons (8, 10). Previous works (16), as well as the current findings, seem to suggest that specific characteristics such as parental PTSD is what enabled the process of intergenerational transmission.

### Limitations and Future Studies

These data should be examined in context of the following limitations. First, we employed a convenience sample which was biased towards academic education and elevated socioeconomic status in a cross-sectional design. Yet, such a sample was also advantageous. Namely, the current study which in contrast to previous works did not specifically select participants from Holocaust organizations, thus likely leading to a less biased sample where obtaining effects is more difficult (10, 64). Given the cross-sectional design, we apply caution regarding causality; at the very most, the significant mediated pathway suggests a possible mechanism that still requires longitudinal verification. Moreover, the sample may not have been large enough to enable clear comparisons of paternal and maternal PTSD. In principal, we also found it difficult to collect data from OHS whose both parents are alive and sufficiently healthy to rate their PTSD levels. Hence, due to this limitation, we could not examine the interaction between paternal and maternal PTSD. Previous studies show that maternal exposure (13) or maternal PTSD (19) may have stronger effects on offspring, whereas other studies found paternal effects as well (23). This issue should be further investigated in the context of offspring successful aging with larger groups of OHS whose Holocaust survivor parents are suffering from Holocaust related PTSD Fourth, our measures of parental PTSD and successful aging were obtained *via* self-report while the interviewers were seminar students. Future studies should include professional interviewers and use a psychiatric assessment of parental PTSD. Other measures, such as biological indices of offspring physical health, which can further increase clarification of result interpretation, were also not included.

### Practical Implications

These data indicate the importance of an interdisciplinary approach. Namely, there might be several underlying mechanisms involved in subsuming the link between parental PTSD and offspring’ successful aging. These processes may be instrumental both in preventive and reactive interventions aimed to increase OHS successful aging. As noted above, the model enables several interventions, each geared at a different model phase, for example, psychotherapy aimed at alleviating the parental child syndrome, or trauma therapy that may reduce secondary traumatization, or therapies designed for depression which may relieve depression symptom levels. OHS successful aging may be further enhanced through preventive programs that may include focusing on specific OHS groups who may be at higher risk for late-life physical health impairments, such as those with parent–child role reversal, high levels of secondary traumatization, and those with elevated depression symptoms. Applying such a focused approach may enable the developing of a campaign to highlight the value of psychosocial interventions as well as preventive activities (e.g., exercise, performing common screening tests) (64). Moreover, more reactive interventions can address successful aging issues and focus on negative aging perceptions, especially in light of data demonstrating that favorable age perceptions are beneficial to future functioning (65).

## Data Availability Statement

The datasets generated for this study are available on request to the corresponding author.

## Ethics Statement

The studies involving human participants were reviewed and approved by Bar Ilan Ethics Committee. The patients/participants provided their written informed consent to participate in this study.

## Author Contributions

Both authors took part in planning the theoretical and conceptual basis for the study. YH wrote the first draft of the introduction and discussion. AS performed the statistical analyses and wrote the first draft of the results. Both authors took part in critically reviewing and editing the manuscript.

## Conflict of Interest

The authors declare that the research was conducted in the absence of any commercial or financial relationships that could be construed as a potential conflict of interest.

## Footnotes

1 According to reports by te Ministry of Social Affairs amd Social Services (2016).
